# The B-type natriuretic peptide of the Congo and Timneh grey parrot

**DOI:** 10.1007/s11259-021-09813-3

**Published:** 2021-07-19

**Authors:** Anja Hennig, L. Mohr, M. Fehr, M. Legler

**Affiliations:** 1 Clinic for Small Mammals, Reptiles and Birds, University of Veterinary Medicine Hannover, Foundation, Bünteweg 9, 30559 Hannover, Germany; 2 Clinic for Poultry, University of Veterinary Medicine Hannover, Foundation, Bünteweg 9, 30559 Hannover, Germany

**Keywords:** Brain natriuretic peptide, Psittacus, Heart, Cardiovascular diseases

## Abstract

In captivity, cardiovascular diseases are common in grey parrots. The diagnosis of these diseases in living birds is difficult, and new diagnostic possibilities would be desirable. The heart is an important endocrine organ in which cardiomyocytes synthetise B-type natriuretic peptide (BNP) and release it into the bloodstream. This hormone has a significant role in cardiovascular and body fluid regulation. The blood concentration of BNP is used in human medicine and small animal medicine as a diagnostic tool in the identification of heart diseases and as a prognostic marker for the risk of mortality. The nucleotide and amino acid sequence of BNP was described in Congo (n = 4) and Timneh (n = 3) grey parrots by PCR after RNA isolation from the atria and ventricles. The results showed a high similarity between the nucleotide sequences of the grey parrots’ BNP and the already known sequence of this hormone in chickens. The amino acid sequence of the mature peptide region is consistent in these three species. BNP plasma concentration could be a possible blood parameter for identifying clinically manifest cardiovascular diseases in grey parrots as it is in other species.

## Background

Grey parrots (*Psittacus erithacus* and *Psittacus timneh*), common pet birds and an endangered species in the wild, are frequently represented in clinical avian consultations (Pees 2004). Dyspnoea and reduced performance are common reasons for presenting grey parrots at the veterinarian. The avian medicine practitioner is constantly confronted with the task of differentiating dyspnoea caused by diseases of the respiratory system, e.g. aspergillosis, from dyspnoea caused by pathological changes in the cardiovascular system. Arteriosclerosis and related clinical signs, especially secondary heart insufficiency, are quite common in grey parrots (Beaufrere et al. 2013).

The clinical examination of the heart is often limited due to the anatomy and physiology of birds, especially in small birds (Pees et al. 2006). To date, the diagnosis of diseases of the cardiovascular system in living birds is still difficult and mostly based on imaging methods, such as radiography and ultrasonography (Bavelaar and Beynen 2004; Pees et al. 2006). The plasma concentrations of the muscle enzymes, creatine kinase (CK), aspartate aminotransferase (AST) and lactate dehydrogenase (LDH), are also not sensitive enough to diagnose a heart disease (Fudge 2000). Further examination techniques and biomarkers are needed to diagnose and characterise cardiovascular diseases.

The heart is an important endocrine organ as it secretes the brain natriuretic peptide (B-type natriuretic peptide, BNP). This hormone was first isolated from the brain of a pig (Sudoh et al. 1988), which led to the name although, as later found out, its concentration is much higher in the heart (Mukoyama et al. 1991). BNP belongs to the natriuretic peptide family, which members all share a 17 amino-acid ring structure built by an intramolecular disulphide bond. The prepropeptide is synthetised in the cardiomyocytes from which pro-BNP is formed by cleavage within the cells. During release into the blood stream, the biologically active form (mature peptide region) arises when the amino-terminal-pro-BNP (NT-pro-BNP) is separated from the pro-BNP so that both peptides can be found in the circulation (Akizuki et al. 1991).

In birds, BNP seems to be the primary natriuretic peptide, even though a C-type-natriuretic peptide (CNP) has been isolated from the heart (Takei et al. 2011; Trajanovska et al. 2007). In contrast, there is also an A-type natriuretic peptide (atrial natriuretic peptide; ANP), isolated from the heart in mammals, which could not be found in birds (Trajanovska and Donald 2008).

By secreting BNP, cardiomyocytes take part in the regulation of the cardiovascular and fluid system of the body. The expression depends on the pressure loading of the cardiac wall (de Bold 1985; Gray 1993). Once released into the blood stream, BNP takes effect on the kidneys causing natriuresis and diuresis, which result in reduced blood pressure. It also works as an antagonist of the renin–angiotensin–aldosterone-system (Gray 1993; Gray et al. 1991). These effects are mediated through different natriuretic peptide receptors (NPR) that catalyse the transformation of guanosintriphosphate into the second messenger cyclic guanosine monophosphate (cGMP). In birds, there seem to be three different NPRs, with BNP binding mostly to the NPR-A receptor (Chinkers and Garbers 1989; Suga et al. 1992; Trajanovska et al. 2007). Afterwards, cGMP can be found in the blood plasma because the major part of it leaves the cells (Hamet et al. 1984).

In humans, the blood concentration of BNP is increased in patients with cardiac diseases due to mechanical stimuli (de Bold et al. 1996). Therefore, the blood concentration of the natriuretic peptide and its N-terminal fragment (NT-pro-BNP) is used in human medicine as a diagnostic tool in patients presenting with acute dyspnoea for the identification of heart diseases and differentiation from primary diseases of the respiratory system (Torres-Courchoud and Chen 2016). It can also be used as an efficient prognostic marker for the risk of future cardiac events and mortality (Santaguida et al. 2014).

Similarly, for clinical small animal medicine, a NT-pro-BNP test is currently used for dogs and cats and there are several studies evaluating the use of BNP in dogs and cats (Baisan et al. 2016).

However, in bird species, the amino acid sequence of BNP is known for some poultry species such as chickens and pigeons (Miyata et al. 1988; Trajanovska and Donald 2008). No studies have been published that investigate BNP in psittacine birds and many other bird species.

In this study, we investigated the nucleotide and resulting amino acid sequence of B-type-natriuretic peptide in grey parrots as a first step towards a possible use of the natriuretic peptide as a diagnostic tool in avian medicine.

## Material and methods

### Animals

In this study, the hearts of seven grey parrots (*P. erithacus*: *n* = 4; *P. timneh*: *n* = 3) were examined. In addition to the samples from the grey parrots, samples from a racing pigeon (*Columba livia* f. domestica) and chicken heart (*Gallus gallus* f. domestica) were also examined to serve as controls.

All animals were presented as patients in the clinic for small mammals, reptiles and birds in Hannover. They died or had to be euthanised because of different diseases and were donated by the owner to the clinic for scientific purposes. The hearts were isolated from the body within 24 h after death and stored at -70 °C. Equal-sized samples of left and right atria and ventricles were taken directly before isolation of the RNA, adding up to a total amount of approximately 50 mg.

### RNA isolation

The total RNA was isolated from the hearts using the MasterPureTM RNA Purification Kit (Epicentre by Illumina, San Diego, California, USA), including a DNase treatment against contamination, and again stored at -70 °C for up to a month until examination. RNA-Concentrations were measured with the NanoDrop™ 1000 Spectrophotometer (Thermo Fisher Scientific Inc., Waltham, Massachusetts, USA), showing a range from approximately 500 to 2500 ng/µl. On the day of the PCR 8 µl of RNA, containing 4 to 20 µg of total RNA, were converted into cDNA using the SuperScript®III First Strand Synthesis System for RT-PCR (InvitrogenTM by Thermo Fisher Scientific Inc., Waltham, Massachusetts, USA).

### PCR

A PCR was performed with a forward (5’- GAGAGGGACCTGAAGAGACAT- 3’) and a reverse (5’- CGTCTTGGGAGGAACAGGTAC—3’) gene-specific primer, published and designed to detect BNP in pigeons (Trajanovska et al. 2007). After an initial denaturation at 94 °C for 2 min, 35 cycles of PCR were performed, each consisting of a denaturation at 94 °C for 30 s, annealing at 60 °C for 30 s and extension at 72 °C for 45 s. A final extension at 72 °C for 2 min was subsequently performed (Trajanovska 2008).

### Gel electrophoresis

**Fig. 1:**
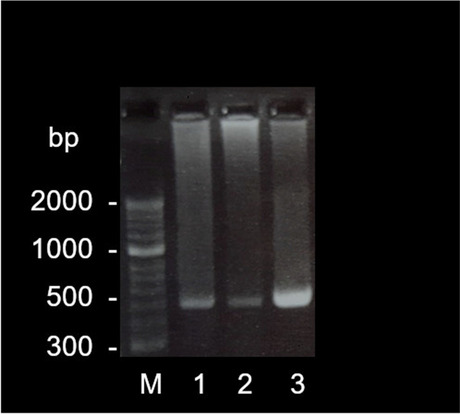
Image of the PCR product (base pairs, bp) of a pigeon (1), two Congo grey parrots (2, 3) and the marker (M) on the GelRed®-stained 2% agarose gel after gel electrophoresis

The products of the PCR were passed through gel electrophoresis on a 2% agarose gel (Biozym Scientific GmbH, Hessisch Oldendorf, Germany) at 120 V and 400 mA for 35 min (Bio-Rad Laboratories GmbH, Feldkirchen, Germany; Fig. 1) using a DNA marker covering a range from 50 base pairs (bp) up to 2 kbp (Biozym Scientific GmbH, Hessisch Oldendorf, Germany). Afterwards, the PCR bands were cleaned up with the Nucleo-Spin® Gel and PCR Clean-up (Macherery-Nagel GmbH & Co. KG, Düren, Germany) and bidirectionally sequenced in an external laboratory by Sanger Sequencing (Microsynth Seqlab GmbH, Göttingen, Germany).

### Nucleotide and amino acid sequences analysis

The received forward and reverse sequences were aligned using BioEdit® (Version 7.2.5. (Hall 2001)) and translated into amino acid sequences. Subsequently both the nucleotide and amino acid sequences were compared to known BNP sequences from birds with GenBank® and BioEdit®.

## Results and discussion

The PCR products isolated from the Congo and Timneh grey parrot hearts consisted of sequences of approximately 480 bp (Fig. 1) and in each of the samples, the mature peptide region of BNP could be found.

**Fig. 2:**
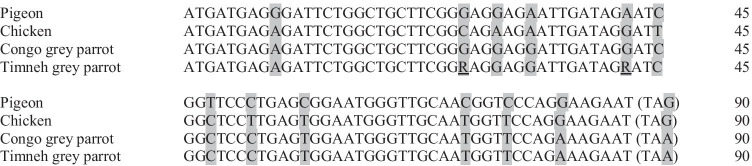
Nucleotide sequence of the mature peptide region of B-type-natriuretic peptide in pigeon, chicken, Congo and Timneh grey parrots consisting of 90 bases each. The stop codon is shown in brackets. The nucleotides that differ within the species are shaded. The nucleotides distinguished between the Timneh grey parrots are underlined

The nucleotide sequence of the mature peptide region was concurrent in all examined Congo grey parrots. In the three Timneh grey parrots, there were substitutions of one nucleotide at the third position between each examined animal (Timneh 1: identical to Congo Grey Parrot; Timneh 2: adenine instead of guanine at base number 37; Timneh 3: adenine instead of guanine at base number 42). Both Congo and Timneh grey parrot mature peptide region nucleotide sequences were similar to those found in chickens and pigeons (Fig. 2). The sequence of Congo grey parrots and the chicken sequence showed a similarity of 94%, while that of Congo grey parrot and pigeon were 91% identical. The Timneh grey parrot sequence shared 93% of the nucleotides with the chicken sequence and 90% with the pigeon nucleotide sequence.

**Fig. 3:**
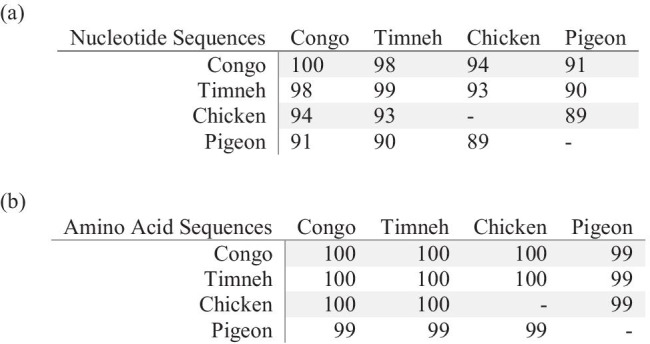
Compilation of the similarity of the nucleotide and amino acid sequence of the mature peptide region of BNP in percent in the examined species. Since only one individual was examined in chicken and pigeon, those fields remain blank

The chicken and pigeon nucleotide sequences themselves showed a similarity of 89% (all percentages based on the mature peptide region excluding the stop codon) (Fig. 3a).

**Fig. 4:**
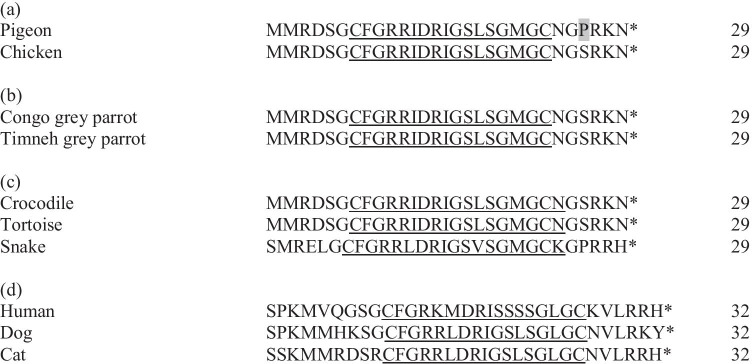
The amino acid sequence of the BNP mature peptide region of (**a**) the established species chicken and pigeon, (**b**) the grey parrot species found out in this study, (**c**) reptiles and (**d**) humans, dogs and cats. The 17-amino-acid ring structure is underlined. The asterisk marks a stop codon. The amino acid in the pigeon that differs from the sequence found in the grey parrot is shaded. The grey parrot sequence shows an aberration of 3,4% to the one from pigeon. GenBank accession numbers: NM_204925.1 Gallus gallus natriuretic peptide A (NPPA); NM_001282844.1 Columba livia natriuretic peptides A-like (BNP); AY398687.1 Crocodylus porosus B-type natriuretic peptide; AY433953.1 Chelodina longicollis B-type natriuretic peptide (BNP); EF667966.1 Pseudonaja textilis B-type natriuretic peptide precursor (BNP); NM_002521.3 Homo sapiens natriuretic peptide B (NPPB); XP_013966931.1 Canis lupus familiaris natriuretic peptides B; NM_001009244.1 Felis catus natriuretic peptide B (NPPB)

The amino acid sequence of the mature peptide region, containing 29 amino acids, was concurrent in all examined grey parrots and there was a substitution of only one single amino acid from the already known sequence of the pigeon (serine vs. proline) while it was identical to the one known for chickens (Figs. 3b, 4a, b).Compared to the mature peptide region of reptiles, BNP of grey parrots is identical to sequences from tortoise and crocodile, whilst the sequence known from snakes differs at several positions (Fig. 4c). The mature peptide region known from mammals is not only longer (32 instead of 29 amino acids) but also shows major differences in the sequence itself (Fig. 4d). The results show that the perception that the structure of the B-type natriuretic peptide has been highly conserved during the evolution of birds (Takei et al. 2011) could be confirmed. They also explain the finding that synthetic chicken BNP leads to a dose-dependent increase of the blood plasma concentration of the natriuretic peptide second messenger guanosine monophosphate in Congo grey parrots (Legler 2009), since the mature peptide region of BNP in Grey Parrots was consistent with the ones known from chicken. That study also investigated cGMP concentrations in healthy and diseased grey parrots with clinically manifest heart diseases due to atherosclerosis. There were no significant differences between the cGMP blood plasma concentrations of the parrots with symptoms of cardiovascular diseases and clinically healthy individuals, which led to the conclusion that cGMP is not a suitable blood parameter for diagnosing heart diseases in grey parrots.

Case reports and studies describing diseases of the cardiovascular system exist for many avian species. Reports include different species of parrots and raptors as well as some other birds (Beehler et al. 1980; Fischer et al. 2005; Legler et al. 2017; Mitchell et al. 2008; Pees et al. 2006).

The BNP nucleotide sequences for some bird species predicted by automated computational analysis can be found at GenBank®. Most of those sequences show a high similarity within the range of the mature peptide region compared to the one found in this study for the grey parrots if translated into amino acid sequences.

In summary, BNP might be an eligible candidate for developing a diagnostic tool for cardiovascular diseases to improve the medical care for birds in captivity. Further studies are necessary to investigate whether BNP could be a helpful blood parameter for diagnosing and monitoring heart diseases in grey parrots as it is in humans. Research on the BNP sequence of other bird species is required to form the basis for potentially extending that possible tool to other bird species.

## Data Availability

Not applicable
